# Cross-Reactivity of Filariais ICT Cards in Areas of Contrasting Endemicity of *Loa loa* and *Mansonella perstans* in Cameroon: Implications for Shrinking of the Lymphatic Filariasis Map in the Central African Region

**DOI:** 10.1371/journal.pntd.0004184

**Published:** 2015-11-06

**Authors:** Samuel Wanji, Nathalie Amvongo-Adjia, Benjamin Koudou, Abdel Jelil Njouendou, Patrick W. Chounna Ndongmo, Jonas A. Kengne-Ouafo, Fabrice R. Datchoua-Poutcheu, Bridget Adzemye Fovennso, Dizzle Bita Tayong, Fanny Fri Fombad, Peter U. Fischer, Peter I. Enyong, Moses Bockarie

**Affiliations:** 1 Parasites and Vector Biology research unit (PAVBRU), Department of Microbiology and Parasitology, University of Buea, Buea, Cameroon; 2 Research Foundation for Tropical Diseases and the Environment (REFOTDE), Buea, Cameroon; 3 Laboratory of Parasitology and Ecology, Department of Animal Biology and Physiology, University of Yaoundé 1, Yaounde, Cameroon; 4 Centre for Neglected Tropical Diseases (incorporating the Lymphatic Filariasis Support Centre), Liverpool School of Tropical Medicine, Liverpool, United Kingdom; 5 Infectious Diseases Division, Department of Internal Medicine, Washington University School of Medicine, St. Louis, Missouri, United States of America; London School of Hygiene and Tropical Medicine, UNITED KINGDOM

## Abstract

**Background:**

Immunochromatographic card test (ICT) is a tool to map the distribution of *Wuchereria bancrofti*. In areas highly endemic for loaisis in DRC and Cameroon, a relationship has been envisaged between high *L*. *loa* microfilaria (Mf) loads and ICT positivity. However, similar associations have not been demonstrated from other areas with contrasting levels of *L*. *loa* endemicity. This study investigated the cross-reactivity of ICT when mapping lymphatic filariasis (LF) in areas with contrasting endemicity levels of loiasis and mansonellosis in Cameroon.

**Methodology/Principal Findings:**

A cross-sectional study to assess the prevalence and intensity of *W*. *bancrofti*, *L*. *loa* and *M*. *perstans* was carried out in 42 villages across three regions (East, North-west and South-west) of the Cameroon rainforest domain. Diurnal blood was collected from participants for the detection of circulating filarial antigen (CFA) by ICT and assessment of Mf using a thick blood smear. Clinical manifestations of LF were also assessed. ICT positives and patients clinically diagnosed with lymphoedema were further subjected to night blood collection for the detection of *W*. *bancrofti* Mf. Overall, 2190 individuals took part in the study. Overall, 24 individuals residing in 14 communities were tested positive by ICT, with prevalence rates ranging from 0% in the South-west to 2.1% in the North-west. Lymphoedema were diagnosed in 20 individuals with the majority of cases found in the North-west (11/20), and none of them were tested positive by ICT. No Mf of *W*. *bancrofti* were found in the night blood of any individual with a positive ICT result or clinical lymphoedema. Positive ICT results were strongly associated with high *L*. *loa* Mf intensity with 21 subjects having more than 8,000 *L*. *loa* Mf ml/blood (Odds ratio = 15.4; 95%CI: 6.1–39.0; p < 0.001). Similarly, a strong positive association (Spearman’s rho = 0.900; p = 0.037) was observed between the prevalence of *L*. *loa* and ICT positivity by area: a rate of 1% or more of positive ICT results was found only in areas with an *L*. *loa* Mf prevalence above 15%. In contrast, there was no association between ICT positivity and *M*. *perstans* prevalence (Spearman’s rho = - 0.200; p = 0.747) and Mf density (Odds ratio = 1.8; 95%CI: 0.8–4.2; p = 0.192).

**Conclusions/Significance:**

This study has confirmed the strong association between the ICT positivity and *L*. *loa* intensity (Mf/ml of blood) at the individual level. Furthermore, the study has demonstrated that ICT positivity is strongly associated with high *L*. *loa* prevalence. These results suggest that the main confounding factor for positive ICT test card results are high levels of *L*. *loa*. The findings may indicate that *W*. *bancrofti* is much less prevalent in the Central African region where *L*. *loa* is highly endemic than previously assumed and accurate re-mapping of the region would be very useful for shrinking of the map of LF distribution.

## Author Summary

Mapping of lymphatic filariasis (LF) caused by *W*. *brancrofti* is usually done by employing a rapid diagnostic test that permits the detection of worm antigen in daytime blood. This is sometimes combined with a thick blood film (TBF) for microscopic examination, as confirmatory tool for detecting *W*. *bancrofti* Mf in peripheral night blood. During recent epidemiological surveys using immunochromatographic card test (ICT) to map LF in areas highly endemic for loiasis, positive card tests were observed in individuals’ amicrofilaremic for *W*. *bancrofti* during night TBF examination, as well as by parasite DNA detection. The possibility of ICT cross-reacting with *L*. *loa* antigen was envisaged, but so far associations between ICT positivity and *L*. *loa* endemicity levels and loads of Mf in day blood have not yet been established. Moreover, *M*. *perstans* another filaria with blood dwelling Mf, that is often sympatric with *L*. *loa*, could contribute to the observed ICT cross-reactivity. The authors investigated the cross-reactivity of ICT in areas with contrasting endemicity levels of *L*. *loa* and *M*. *perstans* in Cameroon. Results incriminated *L*. *loa* as the major confounder in ICT cross-reactivity, with significant association between ICT positivity and loiasis both at individual level (load of Mf/ml of blood) and endemicity level (Mf prevalence). *M*. *perstans* displayed no association with ICT positivity. The findings raised concerns about the specificity of the whole blood ICT used for LF mapping in loiasis co-endemic areas. The development of an algorithm for LF mapping in loiasis co-endemic areas will be important to validate the LF map obtained using ICT in Central Africa.

## Introduction

Lymphatic filariasis (LF) is a chronic, debilitating vector-borne disease that affects about 68 million people in 73 countries in tropical and subtropical areas of Asia, Africa, the Western Pacific and some areas of the Americas. The disease is caused by the filarial parasites *Wuchereria bancrofti*, *Brugia malayi* and *B*. *timori*, which are transmitted by *Culex*, *Anopheles* and *Mansonia* mosquitoes [1]. In Africa, about 44 million people are currently estimated to be infected with *W*. *bancrofti* [2]. While no human *Brugia* species occurs in Africa, the geographic distribution of *W*. *bancrofti* overlaps considerably with that of the other four African filarial species [3–5]. With its diverse bio-ecological zones, Cameroon is one of the few countries world-wide that is endemic for five human filarial species: *W*. *bancrofti* microfilariae (Mf) mainly in night blood, *Mansonella perstans* and *Loa loa* Mf mainly in day blood, and *Onchocerca volvulus* and *Mansonella streptocerca* Mf in the skin [6].

The presence of circulating Mf in peripheral blood is a prerequisite for the transmission of LF. Elimination efforts of LF therefore focus on the interruption of transmission by decreasing the prevalence of persons with Mf in the population. In 1997, the World Health Assembly targeted LF for elimination through a strategy of mass drug administration (MDA). In Africa where onchocerciasis is co-endemic a single-dose, annual administration of ivermectin (donated by Merck & Co., Rahway, NJ) combined with albendazole (donated by GlaxoSmithKline, Bentford, UK) is recommended for reducing Mf [7,8]. Henceforth, WHO recommended to provide MDA to the entire population at risk of infection in areas with LF prevalence of 1% or higher [7,9–11]. However, accurate mapping of the distribution of infection is crucial for implementation as well as for monitoring and evaluation of the MDA programme.

Historically, detection of Mf by microscopy has been used for the identification of endemic areas. However, parasitological methods to detect Mf require night blood and are time consuming, cumbersome and insensitive [12–14]. Circulating filarial antigen (CFA) tests are now considered the method of choice for the detection of *W*. *bancrofti* infection [15]. CFA can be detected in day blood, and also in individuals that harbour adult worms, few or no Mf. A laboratory-based and two rapid diagnostic filarial antigen tests are now commercially available, and detect a similar epitope. Og4C3 ELISA is a laboratory test using a monoclonal antibody (MAb) of the IgM class against the bovine parasite *Onchocerca gibsoni*. Although this technique allowed for the identification of *W*. *bancrofti* antigens in serum, plasma and hydrocele fluid [16–19]; it is time consuming, expensive and non-convenient for a rapid assessment of LF endemicity. The Filariasis ICT is a rapid diagnostic test, which uses a MAb (AD12) raised against the dog heartworm *Dirofilaria immitis* to detect circulating *W*. *bancrofti* antigen in blood [15]. The test is easy to perform in community settings, and requires no equipment since it comes in a convenient kit format. The ICT has been used as a major tool for mapping, monitoring and evaluation within the Global Programme to Eliminate of Lymphatic Filariasis (GPELF) [5,10,20–23]. Recently, a more sensitive strip test version of the card test that uses the same major reagents became available, and has been evaluated so far in Liberia, Sri Lanka and Indonesia [24,25].

Elimination of LF and onchocerciasis in western and central Africa where loiasis is co-endemic is a problem, because ivermectin that is used for MDA may cause severe adverse events in individuals with high *L*. *loa* Mf loads [26–29]. Therefore, GPELF has not been implementing MDA in many parts of western and central Africa. More difficulties in the advancement of activities of GPELF in this region, have arisen from the incomplete or inaccurate mapping. The ICT has to be read strictly after a 10 minutes incubation period and longer periods can produce false positive test results [30]. Moreover, another problem has recently been reported exclusively from areas with high *L*. *loa* Mf prevalence in Democratic Republic of Congo (DRC). Bakajika et al. [24] observed cross-reaction of the ICT with individuals that harboured large numbers of *L*. *loa* Mf in night blood, without any evidence for LF infection in the study villages. This cross-reactivity with *L*. *loa* antigen was also described in a *L*. *loa* hyper-endemic area in Cameroon [31]. The absence of bancroftian filariasis was proven using both parasitological, and molecular diagnosis.

During the development phase of the ICT, its specificity was tested extensively using sera from patients with various filarial infections, including loiasis [15]. However, the tested loiasis sera were mainly from expats with low Mf densities. The test was not evaluated in areas highly endemic for loiasis, using sera of individuals with high *L*. *loa* Mf counts. Moreover, the contribution of *M*. *perstans*, another filarial parasite with blood dwelling Mf, to the ICT cross-reactivity was not fully resolved. The present study was designed to re-evaluate LF endemicity as assessed by the ICT in areas with contrasting endemicity rates of loiasis and mansonellosis in Cameroon. Data were analysed with the following considerations: (i) association between *L*. *loa* Mf prevalence and the rate of ICT positivity in areas of contrasting endemicity of loiasis; (ii) association between *M*. *perstans* Mf prevalence and the rate of ICT positivity in areas of contrasting endemicities of mansonellosis; (iii) correlation between *L*. *loa* Mf densities (Mf/ml of blood) and the rate of ICT positivity at the individual level; (iv) Correlation between *M*. *perstans* Mf densities (Mf/ml of blood) and the rate of ICT positivity at the individual level; and (v) threshold of risk of ICT card positivity due to *L*. *loa* and *M*. *perstans* prevalence and intensity.

## Methods

### Study sites

**Fig 1:**
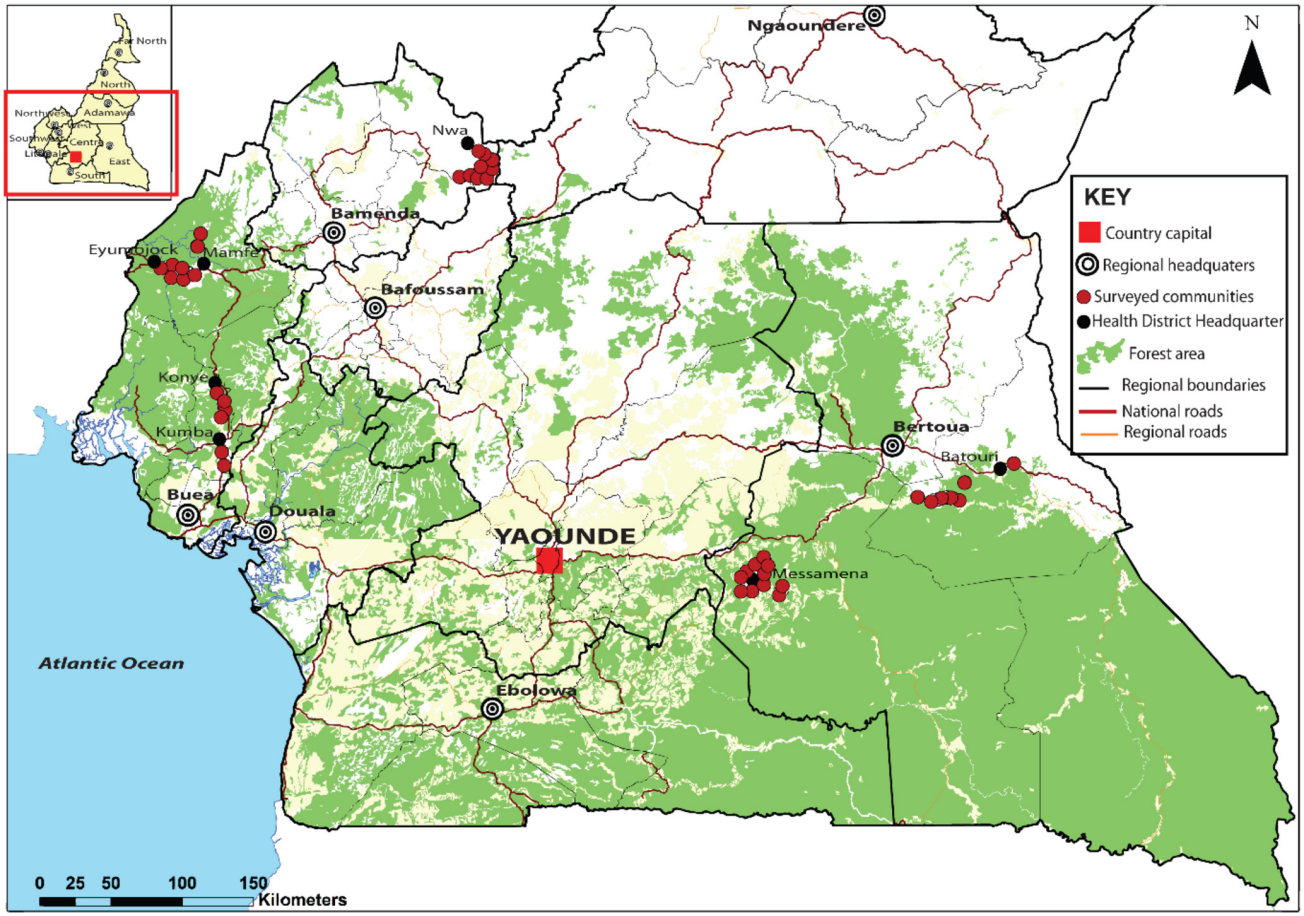
Map of the study area.

Data were collected between March and September 2013 in 42 villages across seven health districts (HDs) in the East (two HDs), North-west (one HD) and South-west (four HDs) regions, located in the Cameroon rainforest belt (Fig 1). The pre-control LF survey data in these study sites showed LF prevalence of 1% and above. These villages are situated in areas of contrasting endemicity of loiasis and mansonellosis. In the East region, Messamena and Batouri HDs are areas of high endemicity for both *L*. *loa* and *M*. *perstan*. The Nwa HD in the North-west is highly endemic for loiasis with very low endemicity for *M*. *perstans*. In the South-west region, four health districts with different profiles of endemicities for loiasis and mansonellosis endemicities were chosen: Kumba-Konye HDs, with low endemicity for both *L*. *loa* and *M*. *perstans* and the Mamfe-Eyumodjock HDs with low endemicity for loaisis and intermediate to high endemicity level for mansonellosis. Except Batouri, which is a naïve HD to ivermectin MDA, the remaining six HDs are under ivermectin MDA for onchocercasis elimination, and the most recent ivermectin MDA took place one to two months before the survey. The ivermectin treatment history in the study area is documented in S1 Table. Ivermectin clears *L*. *loa* Mf and *W*. *bancrofti* Mf at similar rates and it is likely that Individuals with high *L*. *loa* Mf counts did not participate in MDA.

### Study design and population

**Fig 2:**
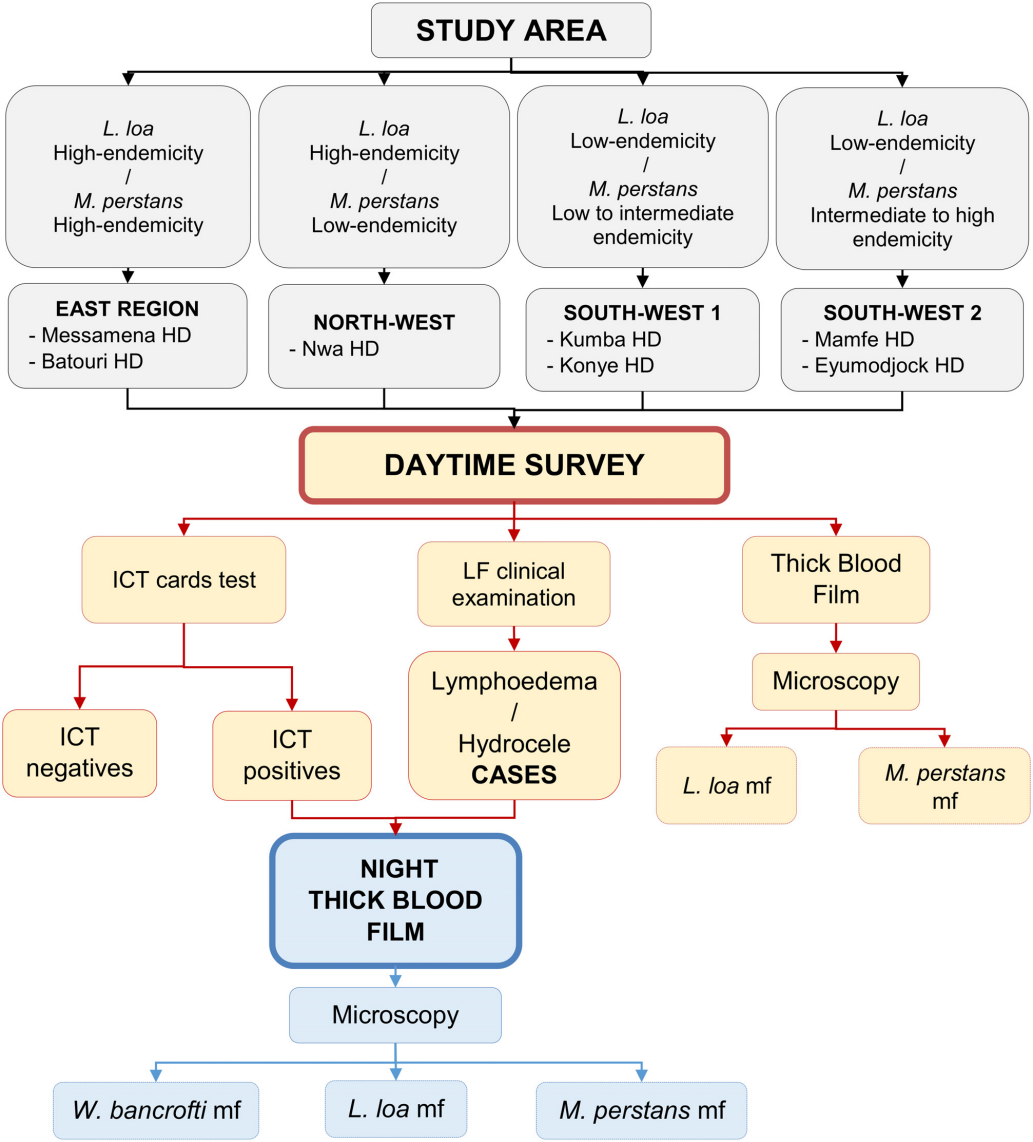
Study design.

Data were collected using a cross-sectional community-based design (Fig 2). In each village, at least 100 residents, both males and females (10 years of age and older) were screened during the day for the presence of *W*. *bancrofti* antigenaemia using the ICT (Alere, Scaborough, ME, USA). Daytime thick blood smear were prepared from each participant to assess their blood dwelling Mf. All study participants were examined for lymphoedema and hydrocele. Individuals tested positive in the ICT and lymphoedema/hydrocele cases, were further submitted to night thick blood film examination for search of *W*. *bancrofti* Mf in the blood.

### Ethical considerations

The protocol used for this study received ethical approval from the Cameroon National ethics committee (CNEC) and administrative approval from the Ministry of Public Health of Cameroon. In the study communities, details were given about potential risks and benefits of the study to the community leaders and study participants. It was explained that participation was voluntary; hence people could withdraw anytime without further obligations. When the head of household or the person involved in the serological survey was well educated and willing to sign documents, investigators provided a written informed consent form which was then signed. However, due to high illiteracy rate and cultural reasons (i.e. signatures or finger prints are linked to elections or court orders), oral informed consent was obtained in many cases from each person participating in the clinical, serological and parasitological examination. The researcher presented the consent information to the participant referring to the bullet points listed on the consent form, and answered any questions he had. The CNEC approved these procedures and if the participant gives consent, it was recorded in the researcher’s notes. If less than 21 years old (age of majority in Cameroon) the verbal assent and permission were obtained from the study participant and legal guardians respectively. The data were analysed and reported, to exclude any directly identifiable information, in order to maintain the anonymity of the participants.

### Clinical examination

Trained medical personnel recruited for this study examined all the participants for lymphoedema. All males were examined for signs of the limb lymphoedema and hydrocele and female for limb lymphoedema [32].

### Circulating filarial antigen examination

The ICT cards were stored at 8°C and carried to the field in polystyrene foam boxes. In each community, the blood from eligible participants was tested in the field by ICT according to the manufacturer’s instructions. Briefly, 100μL of heparinized capillary blood obtained during the day between 10am and 3pm, from each person by finger pricking was applied to the sample application pad of the ICT. The reading was taken strictly after 10 minutes. A single test was performed for each participant.

### Parasitological examination

Diurnal and nocturnal blood collections were performed between 10am-3pm and 10pm-12am, respectively. At daytime, 50μL non-heparinized finger-prick blood was used to assess Mf by thick blood film (TBF). During the night, two separate non-heparinized finger-prick blood samples of approximately 50μL were collected from all ICT positives and all lymphoedema/hydrocele cases to carry out TBF. The blood was drawn onto a microscope slide, allowed to dry and stained with 10% Giemsa using standard procedures [6]. The stained smears were examined using a light microscope at 10× objective for blood dwelling Mf: *L*. *loa* and *M*. *perstans* for daytime TBF and *W*. *bancrofti*, *L*. *loa* and *M*. *perstans* for night TBF. Microfilariae were identified (when need using 40× or 100× objectives), quantified and recorded.

### Statistical analysis

Data were compiled and managed using EpiInfo v3.5.3 (Centers for Disease Control and Prevention, Atlanta, GA) and imported to SPSS v20.0.0 (Armonk, NY: IBM Corp) for analysis. The geometric mean intensity (GMI) of Mf counts was calculated as antilog (∑log(x+1)/n), with "x" being the number of Mf per mL of blood in Mf positive individuals and "n" the number of Mf positive individuals examined. Unless otherwise stated, all statistically significant associations were determined by setting the probability of a Type I error at 5% (α = 0.05). Spearman correlation analysis was carried out between ICT positivity rates and TBF data at both individual and district levels. Chi-square tests were used to compare dichotomous variables. The logistic regression was performed to identify the predictors of ICT positivity in areas where *L*. *loa* and *M*. *perstans* are co-endemic.

## Results

### Study population characteristics

**Fig 3:**
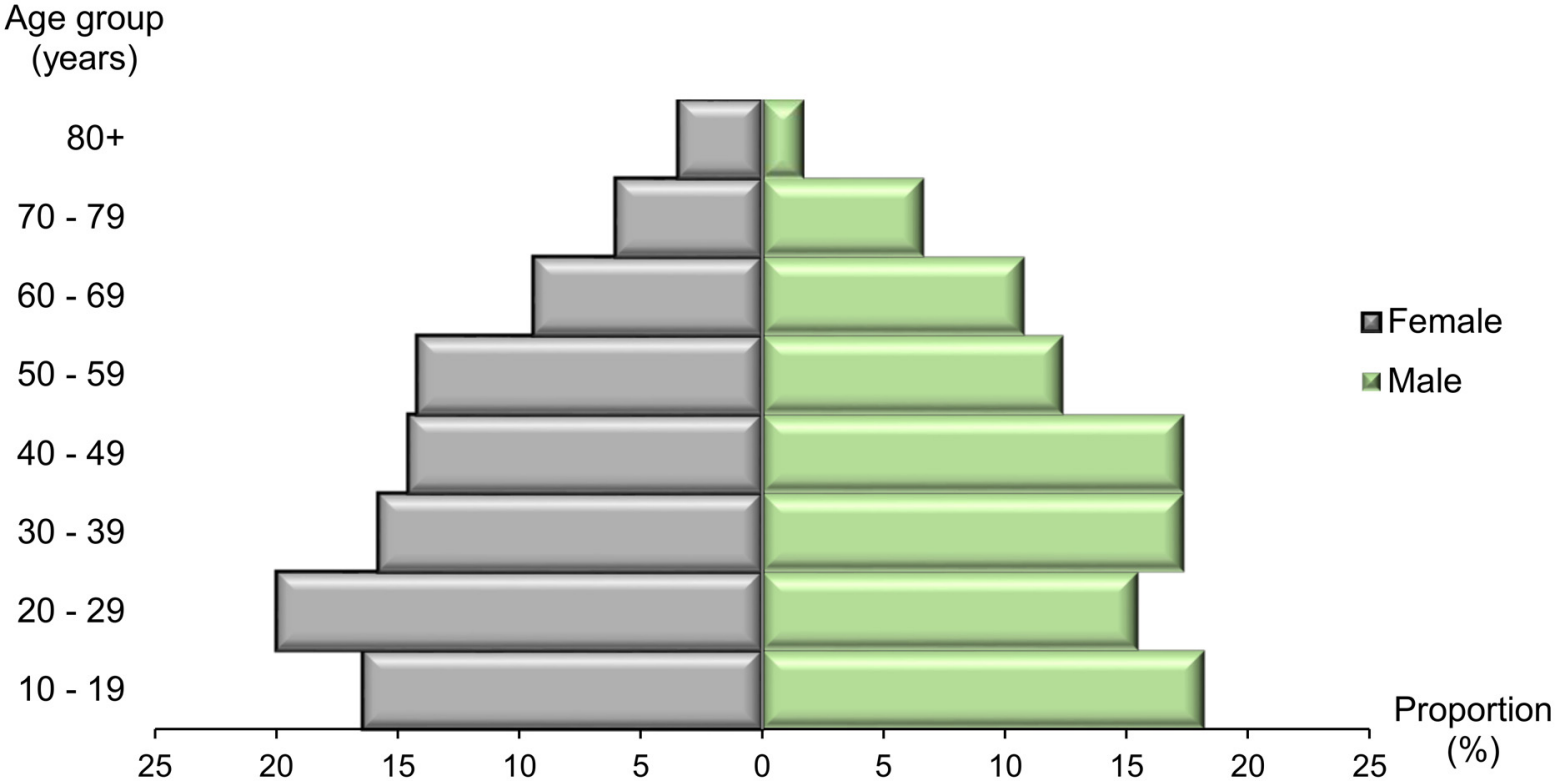
Age distribution of the study population.

Overall, 2190 participants underwent ICT testing. Of these, 48.6% (1065/2190) were male with a mean age of 39.7 ± 19.2 years. The females (1125/2190) had a mean age of 39.6 ± 19.5years. The majority of the participants enrolled were aged between 20 and 59 years (Fig 3).

### Lymphoedema prevalence

Twenty (0.9%) lymphoedema cases were diagnosed in the entire study population, and none of them tested positive by ICT for circulating *W*. *bancrofti* antigen (Table 1). The Northwest site had the highest lymphoedema prevalence (1.8%) followed by East sites (Messamena and Batouri HDs with 1.4% and 0.6% prevalence respectively). Males were slightly more affected by the lymphoedema condition than females (1.3% and 0.5% lymphoedema prevalence respectively), but this difference was not statistically significant (p = 0.055). There was no significant difference in lymphoedema prevalence with respect to age groups (p = 0.176) (Table 2). No single case of hydrocele was observed in males.

**Table 1:** Positive results for antigen detection, clinical manifestations of lymphatic filariasis and microfilaraemia test.

Site	Health district	Community	N° of persons examined	Number of ICT positive (%)	Number of lymphoedema cases (%)	Number of *L*. *loa* Mf positive (%)	*L*. *loa* Mf GMI (Mf/ml)	Number of *M*. *perstans* Mf positive (%)	*M*. *perstans* Mf GMI (Mf/ml)
**EAST**									
	**Batouri**								
		Dem 2	40	0	0	13	1245.8	14	57.4
		Djal	44	0	0	14	2761.1	10	121.0
		Gabaleta	47	0	0	8	1662.1	6	94.2
		Kamba Mieri	60	0	0	16	2777.5	11	81.6
		Konga	36	4	1	14	3220.8	24	201.4
		Ngoulmekong	52	1	0	21	1272.4	29	168.1
		Nguikouassima	63	1	1	15	1067.2	20	78.1
		**Total**	**342**	**6 (1.8)**	**2 (0.6)**	**101 (29.5)**	**1809.4**	**114 (33.3)**	**117.6**
	**Messamena**								
		Aviation	42	1	0	7	797.1	11	46.3
		Bissoua 2	83	0	1	11	412.1	56	189.2
		Doume Village	42	1	0	2	254.6	10	50.9
		Koum	45	0	0	5	519.3	26	94.2
		Labba	34	0	1	2	400.0	27	309.6
		Mayos	75	0	0	6	42.0	39	129.1
		Meba	47	0	3	12	121.0	35	159.6
		Messamena Village	36	0	0	3	559.1	22	147.3
		Nkomzuh	12	0	0	0	-	6	70.5
		Ntollock	52	0	1	11	147.8	14	34.8
		Soleye	43	2	1	6	1642.0	29	164.9
		**Total**	**511**	**4 (0.8)**	**7 (1.4)**	**65 (12.7)**	**277.4**	**275 (53.8)**	**134.7**
	**Total**		**853**	**10 (1.2)**	**9 (1.1)**	**166 (19.5)**	**868.2**	**389 (45.6)**	**129.4**
**NORTH-WEST**									
	**Nwa**								
		Jator	41	1	0	6	2615.7	1	20
		Mbiripkwa	57	2	2	18	652.4	0	-
		Ngomkow	53	1	0	3	3311.1	0	-
		Ngu	59	0	0	14	705.7	0	-
		Nguri	68	2	7	30	1156.1	0	-
		Nking	65	4	0	29	1798.7	0	-
		Ntem	84	0	0	23	494	0	-
		Nwanti	68	2	2	26	1156.7	0	-
		Nwat	59	1	0	6	769.1	0	-
		Sabongari	49	0	0	4	847.4	0	-
	**Total**		**614**	**13 (2.1)**	**11 (1.8)**	**159 (25.9)**	**1022.2**	**1 (0.2)**	**20**
*Table 1 concluded*									
Site	Health district	Community	N° of persons examined	Number of ICT positive (%)	Number of lymphoedema cases (%)	Number of *L*. *loa* Mf positive (%)	*L*. *loa* Mf GMI (Mf/ml)	Number of *M*. *perstans* Mf positive (%)	*M*. *perstans* Mf GMI (Mf/ml)
**SOUTH-WEST 1**									
	**Konye**								
		Baduma	41	0	0	6	372.6	7	64
		Bolo	51	0	0	2	20	8	53.6
		Matondo 2	49	0	0	1	640	4	33.6
		Weme	7	0	0	2	240	4	67.8
		**Total**	**148**	0	0	**11 (7.4)**	**212.3**	**23 (15.5)**	**54.3**
	**Kumba**								
		Ediki	98	1	0	4	787.3	8	36.4
		Mbalangi	101	0	0	9	423.6	2	63.2
		**Total**	**199**	**1 (0.5)**	0	**13 (6.5)**	**512.6**	**10 (5)**	**40.6**
	**Total**		**347**	**1 (0.3)**	0	**24 (6.9)**	**342.2**	**33 (9.5)**	**49.8**
**SOUTH-WEST 2**									
	**Eyumodjock**								
		Ayukaba	45	0	0	2	174.4	6	68.8
		Ebam	54	0	0	3	149.3	1	200
		Mbatop	41	0	0	5	1128	5	64.9
		**Total**	**140**	0	0	**10 (7.1)**	**423.3**	**12 (8.6)**	**73.4**
	**Mamfe**								
		Bache	21	0	0	2	63.2	13	76.8
		Eyanchang	55	0	0	1	7080	1	200
		Kesham	40	0	0	2	2983.2	21	144.4
		Mbakem	40	0	0	1	20	4	73.3
		Taboh	80	0	0	7	456.7	12	34.8
		**Total**	**236**	0	0	**13 (5.5)**	**436.5**	**51 (21.6)**	**83.9**
	**Total**		**376**	0	0	**23 (6.1)**	**430.7**	**63 (16.8)**	**81.8**

No *W*. *bancrofti* Mf were found in ICT positive and lymphoedema cases.

**Table 2:** Gender and age-related prevalence of ICT test card, lymphoedema and diurnal microfilaraemia (TBF).

	N° of persons examined	Number of ICT positive (%)	Number of lymphoedema cases (%)	Number of *L*. *loa* Mf positive (%)	*L*. *loa* Mf GMI (Mf/ml)	Number of *M*. *perstans* Mf positive (%)	*M*. *perstans* Mf GMI (Mf/ml)
*By gender*							
Male	1065	6 (0.6)	14 (1.3)	197 (18.5)	857.1	266 (25.0)	130.3
Female	1125	18 (1.6)	6 (0.5)	175 (15.6)	820.2	220 (19.6)	96.8
*By age group (years*)							
10–19	379	2 (0.5)	3 (0.8)	31 (8.2)	1116.0	64 (16.9)	71.6
20–29	390	6 (1.5)	2 (0.5)	81 (20.8)	981.2	70 (20.3)	69.3
30–39	363	4 (1.1)	2 (0.6)	54 (14.9)	860.4	58 (16.0)	102.3
40–49	349	3 (0.9)	2 (0.6)	70 (20.1)	772.9	72 (20.6)	95.0
50–59	292	2 (0.7)	2 (0.7)	45 (15.4)	590.2	84 (28.8)	158.2
60–69	221	1 (0.5)	4 (1.8)	47 (21.3)	908.7	65 (29.4)	147.8
70–79	139	3 (2.2)	4 (2.9)	28 (20.1)	869.5	46 (33.1)	257.6
80 +	57	3 (5.3)	1 (1.8)	16 (28.1)	583.0	18 (31.6)	159.0

No *W*. *bancrofti* Mf were found in ICT positive and lymphoedema cases.

### Prevalence of positive ICT

Overall prevalence of positive ICT results was 1.1% (24/2190). This prevalence ranged from 0% in the Mamfe-Eyumodjock HD (South-west region) to 2.1% in Nwa HD (North-west region) (Table 1). Two out of seven HDs were found to be positive by ICT with rates greater or equal to 1%: East-Batouri HD (1.8%); Northwest-Nwa HD (2.1%). Among the 42 communities surveyed, 14 had a prevalence of positive ICT ≥ 1%, with 7 of these localized in the Nwa HD in the North-west. One community in the Eastern region (Batouri HD) had a relatively high prevalence (Konga 11.1%). Point prevalence for each surveyed site is shown in S1, S2 and S3 Figs. There were significantly more positive ICT tests in males (1.6%) compared to females (0.6%) (p < 0.05). No significant difference (p = 0.053) was found in antigenaemia prevalence with respect to age groups (Table 2).

### Parasitological findings

**Day blood.** Diurnal blood collection results are also indicated in Table 1. In total, the *L. loa* Mf prevalence was 17.0% (372/2190) with a GMI of 839.5 Mf/ml of blood. The East-Batouri HD (29.5%, 1809.4 Mf/ml GMI) and the North-west (25.9%, 1022.2 Mf/ml GMI) had the highest endemicity levels. In the Southwest 1 (6.9%, 342.2 Mf/ml GMI) and 2 (6.1%, 430.7 Mf/ml GMI) the prevalence of *L. loa* Mf was lower. The point Mf prevalence in each site is shown in S1–S3 Figs. The prevalence of *L. loa* Mf was significantly associated with age (p < 0.001), but no significant difference in *L. loa* infection rate (p = 0.067) was observed between males (18.5%; 857.1 Mf/ml GMI) and females (15.6%; 820.2 Mf/ml GMI).

*Mansonella perstans* Mf displayed a contrasting endemicity pattern in the study area with high prevalence rates observed in the East (45.6%, 129.4 Mf/ml GMI) and Southwest 2 (16.8%, 81.8 Mf/ml GMI). The Southwest 1 (9.5%, 49.8 Mf/ml GMI) and Northwest (0.2%) showed lower prevalence rates. The point prevalence of *M. perstans* Mf in each site is shown in S1–S3 Figs. Males had a significantly (p < 0.05) higher Mf prevalence (25%, 130.3 Mf/ml GMI) compared to females (19.6%, 96.8 Mf/ml GMI) and *M. perstans* infection rates were significantly correlated with age (p < 0.001).

**Night blood.** Out of 43 (23 ICT positives and 20 lymphoedema cases) participants who took part in the night blood collection, none were found positive for *W*. *bancrofti* Mf. Rather, *L*. *loa* and *M*. *perstans* Mf were found in the night TBFs with 41.9% (18/43) and 27.9% (12/43) Mf prevalence respectively.

### Comparison of day and night microfilaraemia

Generally, *L*. *loa* Mf GMI of ICT positives were higher than those of lymphoedema cases (ICT negatives) meanwhile, *M*. *perstans* Mf densities of this last group of individuals were higher as compared to ICT positives. However, *L*. *loa* Mf prevalence slightly decreased between day and night blood examination (*Loa*-day TBF: 17/23; *Loa*-night TBF: 16/23). The *M*. *perstans* Mf rate was not dependent of the time of blood collection (Table 3). *L*. *loa* Mf GMI of ICT-positive individuals decreased from daytime (14366.2 Mf/ml) to the night (1142 Mf/ml), given a reduction coefficient of 12.6, whereas *M*. *perstans* Mf GMI doubled from daytime (100.2 Mf/ml) to the night (202.7 Mf/ml). On the other hand, *L*. *loa* and *M*. *perstans* infection rates of lymphoedema cases (ICT negatives) decreased from day to night, and the Mf densities of both filariae increased between both time points (Table 4).

**Table 3:** Comparison of day and night parasitological indices of ICT positive individuals.

		*L*. *loa* Mf				*M*. *perstans* Mf			
		Day		Night		Day		Night	
	Examined (n)	Mf positive (%)	Mf GMI (Mf/ml)	Mf positive (%)	Mf GMI (Mf/ml)	Mf positive (%)	Mf GMI (Mf/ml)	Mf positive (%)	Mf GMI (Mf/ml)
*Health districts*									
**East**									
Batouri-HD	6	6	12371.5	6	1242.7	6	131	6	265.7
Messamena-HD	4	1	30160	1	280	1	20	1	40
**North-west**									
Nwa-HD	13	10	14591.0	9	1261.8	0	-	0	-
**Total**	**23** *	**17 (73.9)**	**14366.2**	**16 (69.6)**	**1142**	**7 (30.4)**	**100.2**	**7 (30.4)**	**202.7**

* 1 ICT positive (South-west1 site) did not participate to the night blood examination.

**Table 4:** Comparison of day and night parasitological indices in lymphoedema cases.

		*L*. *loa* Mf				*M*. *perstans* Mf			
		Day		Night		Day		Night	
	Examined (n)	Mf positive (%)	Mf GMI (Mf/ml)	Mf positive (%)	Mf GMI (Mf/ml)	Mf positive (%)	Mf GMI (Mf/ml)	Mf positive (%)	Mf GMI (Mf/ml)
*Health districts*									
**East**									
Batouri-HD	2	0	-	0	-	0	-	0	-
Messamena-HD	7	5	161.7	1	1060	6	494.4	5	562.8
**North-west**									
Nwa-HD	11	6	341.1	1	60	0	-	0	-
**Total**	**20**	**11 (55)**	**243.0**	**2 (10)**	**252.2**	**6 (30)**	**494.4**	**5 (25)**	**562.8**

*Loa loa* and *M*. *perstans* Mf periodicity with respect to the Mf load ranges in ICT positives and lymphoedema cases is shown in Tables 5 and 6. Generally, ICT positive individuals with high (> 8,000 Mf/ml) and very high (> 30,000 Mf/ml) *L*. *loa* Mf density were found *Loa* TBF positive both day and night, whereas the low Mf carriers (1–8,000 Mf/ml) were found Mf positive only during the day time.

**Table 5:** *L*. *loa* and *M*. *perstans* Mf periodicity with respect to microfilarial load ranges of ICT positive individuals.

		*L*. *loa* Mf					*M*. *perstans* Mf	
Mf load ranges (Mf/ml)	Examined (n)	Day+/ Night+	Day+/ Night-	Day-/Night-	Day-/Night+	Examined (n)	Day+/ Night+	Day-/Night-
0	6	0	0	5	1	16	0	16
1–8000	3	2	1	0	0	7	7	0
8001–30000	9	8	1	0	0	0	0	0
> 30000	5	5	0	0	0	0	0	0
**Total**	**23**	**15**	**2**	**5**	**1**	**23**	**7**	**16**

**Table 6:** *L*. *loa* and *M*. *perstans* Mf periodicity with respect to microfilarial load ranges of lymphoedema cases.

		*L*. *loa* Mf			Examined (n)	*M*. *perstans* Mf		
Mf load ranges (Mf/ml)	Examined (n)	Day+/ Night+	Day+/ Night-	Day-/Night-		Day+/ Night+	Day+/Night-	Day-/Night-
0	9	0	0	9	14	0	0	14
1–8000	11	2	9	0	6	5	1	0
**Total**	**20**	**2**	**9**	**9**	**20**	**5**	**1**	**14**

### Infection profile of ICT positive individuals and lymphoedema cases

Seventy five percent (18/24) of ICT card positive individuals were *L*. *loa* Mf carriers with 41.7% (10/24) harbouring *L*. *loa* Mf only, and 33.3% (8/24) with both *L*. *loa* and *M*. *perstans* Mf. Six of these individuals had neither *L*. *loa* nor *M*. *perstans* Mf in their day-TBF (Table 7). In this last group of individuals, 3 were from the North-west and 3 from the East-Messamena HD, (areas under ivermectin MDA). ICT positivity was significantly associated to the *L*. *loa* Mf positivity (p < 0.001) with 18 *L*. *loa* Mf positive individuals out of the 24 ICT positives. There was no association between ICT positivity and the positivity of *M*. *perstans* Mf (p = 0.211). Only 8 ICT positives were *M*. *perstans* Mf positive and they were all *L*. *loa* Mf carriers. Lymphoedema cases were all ICT negatives and the majority of them neither have *L*. *loa* nor *M*. *perstans* Mf (Table 8).

**Table 7:** Infection profile of ICT positives cases with respect to *L*. *loa* and *M*. *perstans* infection status using day blood.

		Infection status			
Health district	Examined (n)	*Loa*+/*Pers*+	*Loa+/Pers-*	*Loa-/Pers-*	*Loa-/Pers+*
East-Batouri	6	6	0	0	0
East-Messamena	4	1	0	3	0
Northwest-Nwa	13	0	10	3	0
Southwest 1—Kumba	1	1	0	0	0
**Total**	**24**	**8**	**10**	**6**	**0**

**Table 8:** Infection profile of lymphoedema cases with respect to *L*. *loa* and *M*. *perstans* infection status using day blood.

		Infection status			
Health district	Examined (n)	Loa+/Pers+	Loa+/Pers-	Loa-/Pers-	Loa-/Pers+
East-Batouri	2	0	0	2	0
East-Messamena	7	5	0	1	1
Northwest-Nwa	11	0	6	5	0
**Total**	**20**	**5**	**6**	**8**	**1**

### Individual-level association between ICT positivity and *L*. *loa/M*. perstans Mf intensities

The prevalence of ICT positivity was strongly correlated with Mf loads of *L*. *loa* (Table 9). Only 0.3% of amicrofilaremic individuals were ICT positive. One percent of *L*. *loa* Mf carriers with low Mf load (1–8000 Mf/ml of blood) were ICT positive. Meanwhile, 20.4% and 41.7% of carriers of high (8001–30000 Mf/ml of blood) and very high (> 30,000 Mf/ml of blood) of *L*. *loa* had a positive ICT result respectively.

**Table 9:** Percentage of positive results using ICT card according to the *L*. *loa* load of microfilariae.

	ICT card			
Microfilariae/mL of blood	Positives	%	Negatives	Total
0	6	0.3	1812	1818
1–8000	3	1.0	308	311
8001–30000	10	20.4	39	49
> 30000	5	41.7	7	12

R = 0.438; p < 0.001

This strong association was further confirmed in a logistic regression analysis where it was demonstrated that harbouring *L*. *loa* Mf in the blood was a good predictor for having a positive ICT result (Odds Ratio = 15.4; 95% CI: 6.1–39.0; p < 0.001). The odds of an individual with high Mf load of *L*. *loa* (8001–30000 Mf/ml) to be detected CFA positive by ICT, is 77.4 times higher than an amicrofilaremic individual (OR = 77.4; 95% CI: 26.8–223.7; p < 0.001). The OR becomes extremely high (OR = 215.7; 95% CI: 53.2–874.6; p < 0.001), when an individual harbours very high Mf loads of *L*. *loa* (> 30,000 Mf/ml of blood) (Tables 10 and 11).

**Table 10:** Association between ICT results and *L*. *loa* / *M*. *perstans* positivity rates.

	ICT Results			Statistics		
	Positive	Negative	Total	OR	95%CI	P-value
*L*. *loa* Mf						
***L*. *loa* Mf +**	**18(4.8)**	**354 (95.2)**	**372**	**15.4**	**6.1–39.0**	**< 0.001**
*L*. *loa* Mf -	6 (0.3)	1812(99.7)	1818	-	-	-
*M*. *perstans*						
***M*. *perstans* Mf +**	**8 (1.6)**	**478 (98.4)**	**486**	**1.8**	**0.8–4.2**	**0.192**
*M*. *perstans* Mf -	16 (0.9)	1688 (99.1)	1704	-	-	-

Numbers in brackets are percentages.

**Table 11:** Logistic regression analysis of ICT results according to *L*. *loa* load among the Mf carriers.

	ICT Results			Statistics		
*L*. *loa* Mf load (Mf/ml)	Positive	Negative	Total	OR	95%CI	P-value
1–8,000	3 (1)	308(99)	311	2.9	0.7–11.8	0.128
8,001–30,000	10 (20.4)	39(79.6)	49	77.4	26.8–223.7	< 0.001
> 30,000	5 (41.7)	7(58.3)	12	215.7	53.2–874.6	< 0.000

Numbers in brackets are percentages.

### District-level association between ICT prevalence and *L*. *loa*/*M*. *perstans* Mf prevalence

**Fig 4:**
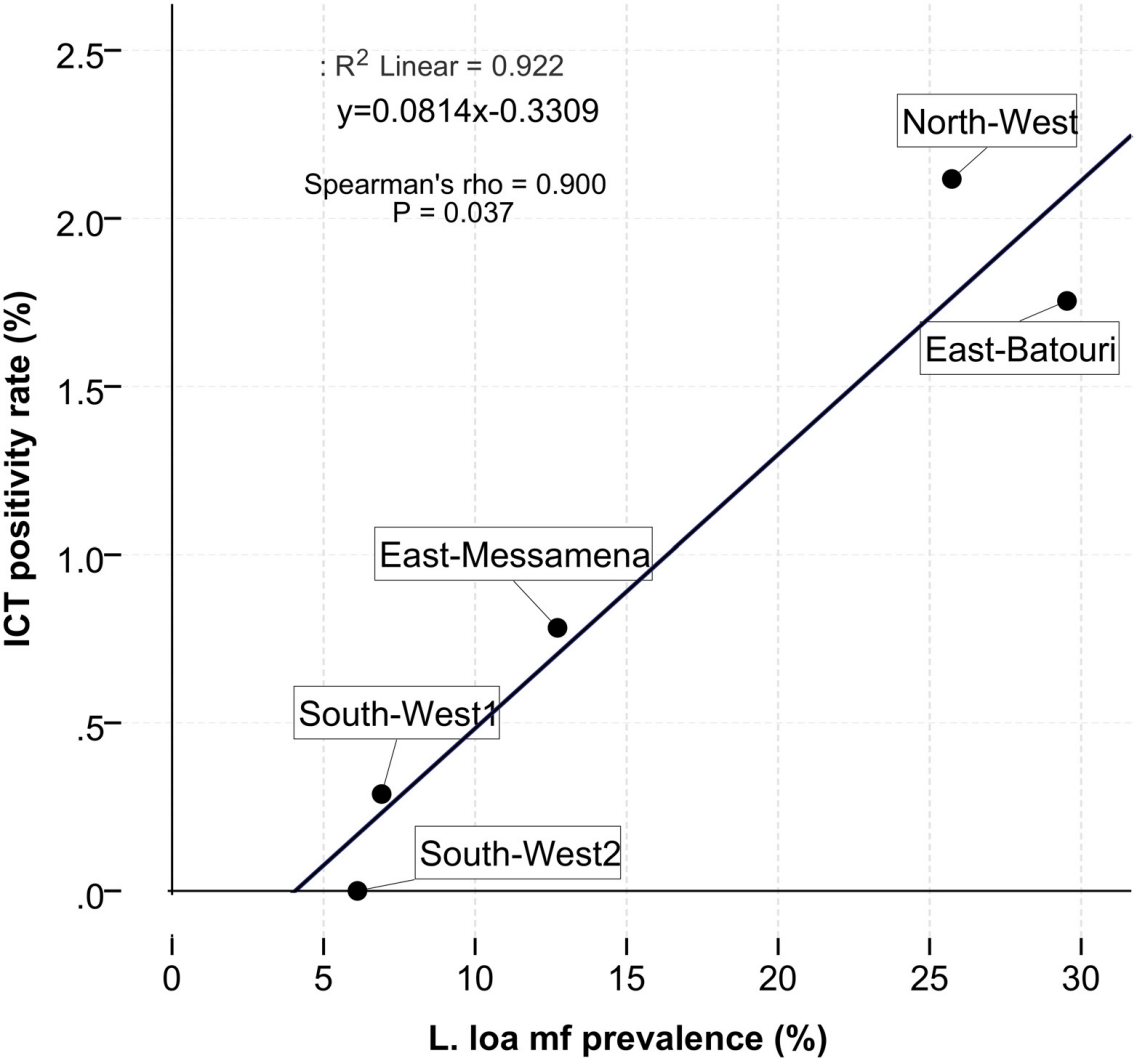
Correlation between ICT positivity rate and *L*. *loa* Mf prevalence.

**Fig 5:**
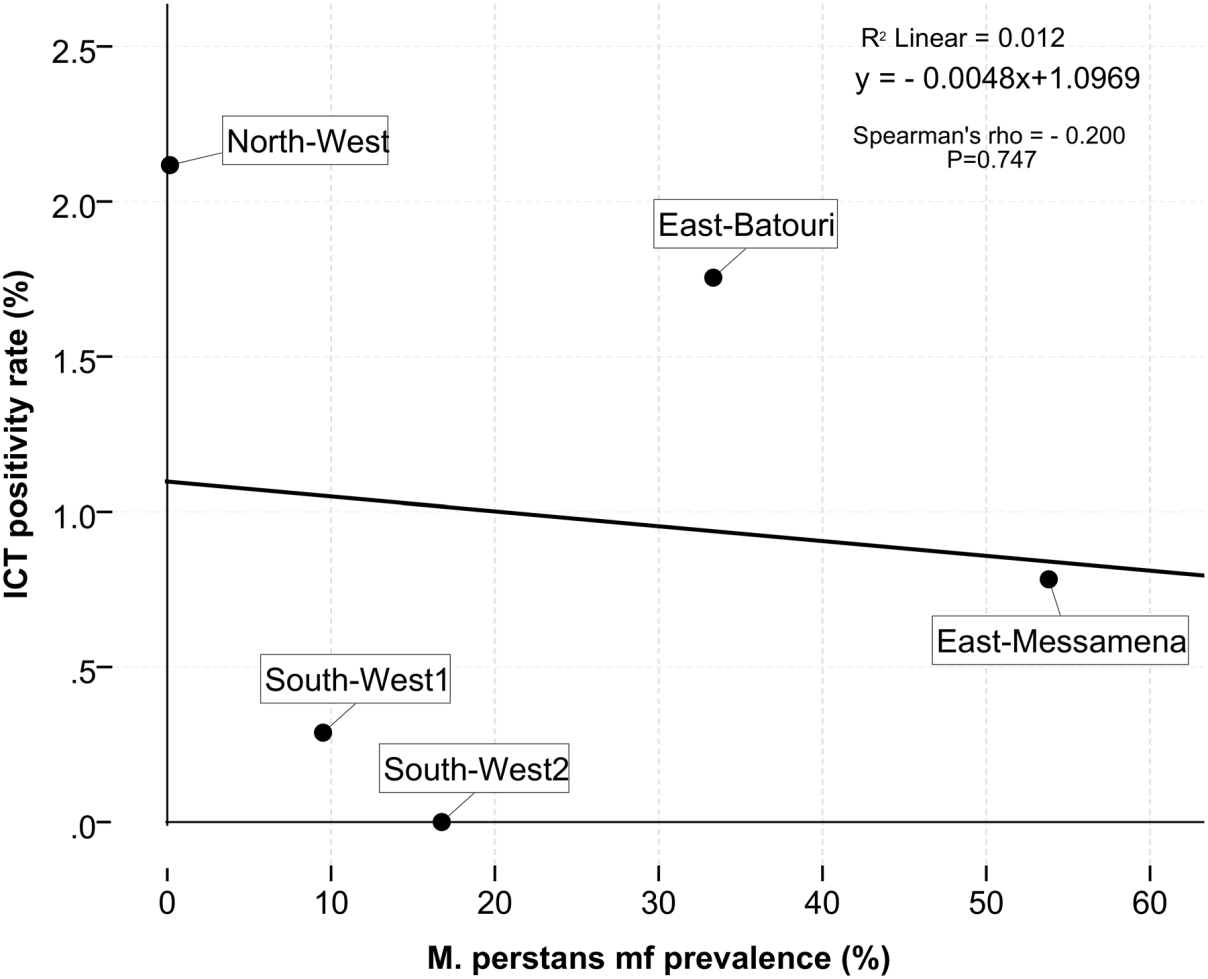
Correlation between ICT positivity rate and *M*. *perstans* Mf prevalence.

The ICT prevalence was strongly correlated (Spearman’s rho = 0.900; p < 0.05) with the endemicity level of loiasis in the different sites as illustrated in Fig 4. ICT positivity was very rare or low in areas where *L*. *loa* Mf prevalence was below 10%. A positive ICT prevalence of ICT up to 0.8% could be seen in areas where the *L*. *loa* Mf prevalence ranged between 10–15%. When prevalence rates of *L*. *loa* Mf were above 25%, rates of more than 1% of ICT positives were detected. In contrast, there was no significant correlation between the prevalence of positive ICT and *M*. *perstans* Mf prevalence (Spearman’s rho = - 0.200; p = 0.747) as illustrated in Fig 5.

## Discussion

The ICT has been extensively used to map LF caused by *W*. *bancrofti* in Asia, Latin America, and North, West and East Africa. Most of these surveys were conducted in loiasis non-endemic areas [20,33–36]. Furthermore, in most areas the ICT was used as sole diagnostic test for routine mapping and was rarely confirmed by additional diagnostic procedures; such as the night TBF and microscopic examination to confirm the presence of *W*. *bancrofti* Mf in the peripheral blood. The present study is the first to evaluate in detail the specificity of the ICT in areas with contrasting endemicity levels of loiasis and mansonellosis in Cameroon.

The Mf results obtained for *L*. *loa* and *M*. *perstans* Mf prevalence were in agreement with previous studies [37–41]. The difference in *L*. *loa* Mf prevalence observed in the East sites can be attributed to the impact of ivermectin MDA on loiasis parasitological indices, since annual community directed treatment with ivermectin (CDTI) is ongoing in Messamena-HD for almost 10 years while Batouri-HD is a CDTI naïve area.

The ICT identified 1.1% (24/2190) *W*. *bancrofti* amicrofilaremic individuals as positive for circulating filarial antigen. The North-west and East sites showed a higher rate of positive ICT (2.1% and 1.2% of ICT positive tests respectively). Night blood examination results revealed the presence of two other blood dwelling Mf, *L*. *loa* and *M*. *perstans* known as endemic in the rainforest domain of Africa. These results are in line with previous ones, where a high sensitivity of ICT at detecting *Loa*-microfilaremic patients was observed in the Democratic Republic of Congo [24]. The only clinical manifestation that may related to LF in the present study was lymphoedema. This has been reported by several investigators as the cause of disability and disfigurement in endemic areas [42–44]. Twenty (0.9%) lymphoedema cases were diagnosed and none of them were tested positive by ICT or found harbouring *W*. *bancrofti* Mf in their night blood. The majority of the lymphoedema cases were found in the North-west site (11/20). Other studies have reported lymphoedema cases of non-filarial origin in the North-west region of Cameroon. This condition (podoconiosis) is caused by irritating micro-particles from the soil [45].

During this study, the infection profile of ICT positives clearly indicates high *L*. *loa* Mf rates (73.9%) compared to a relatively low prevalence rates of *M*. *perstans* Mf (30.4%). More than 65% of *L*. *loa* Mf carriers in this group had high (> 8,000 Mf/ml) and very high (> 30,000 Mf/ml) Mf loads, while *M*. *perstans* Mf had low densities (1–8,000 Mf/ml). On the other hand, low Mf densities and prevalence were observed for the lymphoedema cases. This confirms that lymphoedema is not associated with loiasis or mansonellosis.

A decrease of *L*. *loa* Mf prevalence occurs between day and night TBF Mf carriers with low Mf loads, contrary to high Mf carriers, whose prevalence remains practically the same. *Mansonella perstans* Mf prevalence did not vary between the day and night thick blood smears diagnosis. More studies are necessary to better estimate threshold values of Mf densities at which *L*. *loa* Mf prevalence is not influenced by the time of blood collection.

None of the ICT positives was infected with *M*. *perstans* Mf only. Among the 17 Mf positive individuals, 10 had *L*. *loa* single infections and 8 were co-infected with both *L*. *loa* and *M*. *perstans*. The remaining 6 ICT positive individuals were amicrofilaremic for both parasites. This last group of individuals was recruited from areas of intermediate to high endemicity level of loiasis (East-Messamena and North-west) under implementation of community-directed treatment with ivermectin (9 and 8 rounds of treatment respectively). They might harbour other parasites or stages of filarial species (especially adult worms) responsible of the ICT positivity observed, since the study was carried out few months after the MDA. In *W*. *bancrofti* infections, antigenaemia is common in amicrofilaremic individuals [14]. It is assumed that, the ICT is detecting circulating adult worm antigen, and high Mf levels are usually associated with more adult worms that shed antigen. Further studies to investigate the extent of the ICT card cross-reactivity with different stages of filarial parasites through *in vitro* maintenance may be helpful.

An association between ICT positivity rate and loiasis/mansonellosis prevalence was analysed using spearman correlation analysis. ICT rate was strongly correlated with loiasis endemicity level. Moreover, this model indicated that for *L*. *loa* Mf prevalence greater than 4.3%, there is a possibility to detect ICT positivity related to loiasis. In contrast, no correlation was observed between the ICT positivity rate and *M*. *perstans* Mf prevalence. Henceforth, the logistic regression analysis indicated an association between ICT positivity and *L*. *loa* Mf density. This association was significant for individuals carrying high and very high Mf loads. This observation was previously made by Pion et al. [31] who demonstrated a relationship between high *L*. *loa* Mf density and ICT positivity while mapping LF in area with loiasis in the South region of Cameroon. These observations put together, raise the question whether the ICT can be modified to detect individuals at risk of severe adverse reactions due to high *L*. *loa* Mf densities during ivermectin MDA.

Another implication of our observations is that the LF map in the central African region needs validation taking into consideration the endemicity of *L*. *loa*. Recently, an improved rapid format antigen detection test that uses the same principal reagents as the ICT was developed. This new Alere filariasis test strip is more sensitive compared to the ICT, uses a smaller volume of blood, has a longer shelf time, and will be significantly cheaper [24,25]. This new test has been endorsed by GPELF and will replace the ICT in the near future. The problem of cross-reactivity with loiasis observed with ICT is likely to remain with the new Alere filariasis test strip. The Cameroon LF map based on ICT considerably overlaps the geographic distribution of *L*. *loa*. However, the environmental suitability for LF transmission in Cameroon seems not in good agreement with the LF map based on ICT [46]. Therefore, an algorithm for LF mapping in loiasis co-endemic areas would be helpful to validate the ICT map of LF in Central Africa. Furthermore, with the expansion of MDA for LF elimination in Africa to areas co-endemic for loiasis, it is crucial to select appropriate diagnostic tools for monitoring and evaluation of the elimination program. Transmission assessment surveys based on ICT alone as recommended by WHO may not be suitable for areas co-endemic with *L*. *loa*. Our study has shown the need of confirmatory tests for LF detection to supplement the ICT in areas highly endemic for loiasis.

## Supporting Information

S1 Table Ivermectin treatment history in the study area. IVM: ivermectin.
(PDF)

S1 Fig Point prevalence of *L*. *loa*, *M*. *perstans* and ICT in the east sites. 1. Djal, 2. Gabaleta; 3. Kamba mieri, 4. Nguikouassima, 5. Ngoulemekong, 6. Konga, 7. Dem 2, 8. Ntollock, 9. Doume village, 10. Soleye, 11. Bissoua 2, 12. Mayos, 13. Labba, 14. Meba, 15. Koum, 16. Messamena village, 17. Nkonzuh, 18. Aviation.
(PDF)

S2 Fig Point prevalence of *L*. *loa*, *M*. *perstans* and ICT in the northwest. 19. Ntem, 20. Nwanti, 21. Nguri, 22. Ngu, 23. Nking, 24. Mbiripkwa, 25. Nwat, 26. Sabongari, 27. Jator, 28. Ngomkow.
(PDF)

S3 Fig Point prevalence of *L*. *loa*, *M*. *perstans* and ICT in the southwest sites. 29. Mbakem, 30. Taboh, 31. Ayukaba, 32. Mbatop, 33. Eyanchang, 34. Ebam, 35. Kesham, 36. Bache, 37. Weme, 38. Bolo, 39. Baduma, 40. Matondo, 41. Ediki, 42. Mbalangi.
(PDF)
