# Supplementary material for: Cross-Reactivity of Filariais ICT Cards in Areas of Contrasting Endemicity of Loa loa and Mansonella perstans in Cameroon: Implications for Shrinking of the Lymphatic Filariasis Map in the Central African Region
Source: PLoS Negl Trop Dis. 2015 Nov 6;9(11):e0004184. doi: 10.1371/journal.pntd.0004184 (PMC4636288; doi:10.1371/journal.pntd.0004184)
Supplement: S1 Table — IVM: ivermectin. (PDF) [file pntd.0004184.s001.pdf]

Study sites	Health districts	IVM treatment year started	Number of round of IVM treatment before the study	Number of communities surveyed
South-west 1	Konye<br>Kumba	1999	14	6
South-west 2	Eyumodjock<br>Mamfe	2001	12	8
North-west	Nwa	2004	9	10
East	Messamena	2005	8	11
	Batouri	–	0	7

IVM: ivermectin.
