# Supplementary material for: Cross-Reactivity of Filariais ICT Cards in Areas of Contrasting Endemicity of Loa loa and Mansonella perstans in Cameroon: Implications for Shrinking of the Lymphatic Filariasis Map in the Central African Region
Source: PLoS Negl Trop Dis. 2015 Nov 6;9(11):e0004184. doi: 10.1371/journal.pntd.0004184 (PMC4636288; doi:10.1371/journal.pntd.0004184)
Supplement: S1 Fig — 1. Djal, 2. Gabaleta; 3. Kamba mieri, 4. Nguikouassima, 5. Ngoulemekong, 6. Konga, 7. Dem 2, 8. Ntollock, 9. Doume village, 10. Soleye, 11. Bissoua 2, 12. Mayos, 13. Labba, 14. Meba, 15. Koum, 16. Messamena village, 17. Nkonzuh, 18. Aviation. (PDF) [file pntd.0004184.s002.pdf]

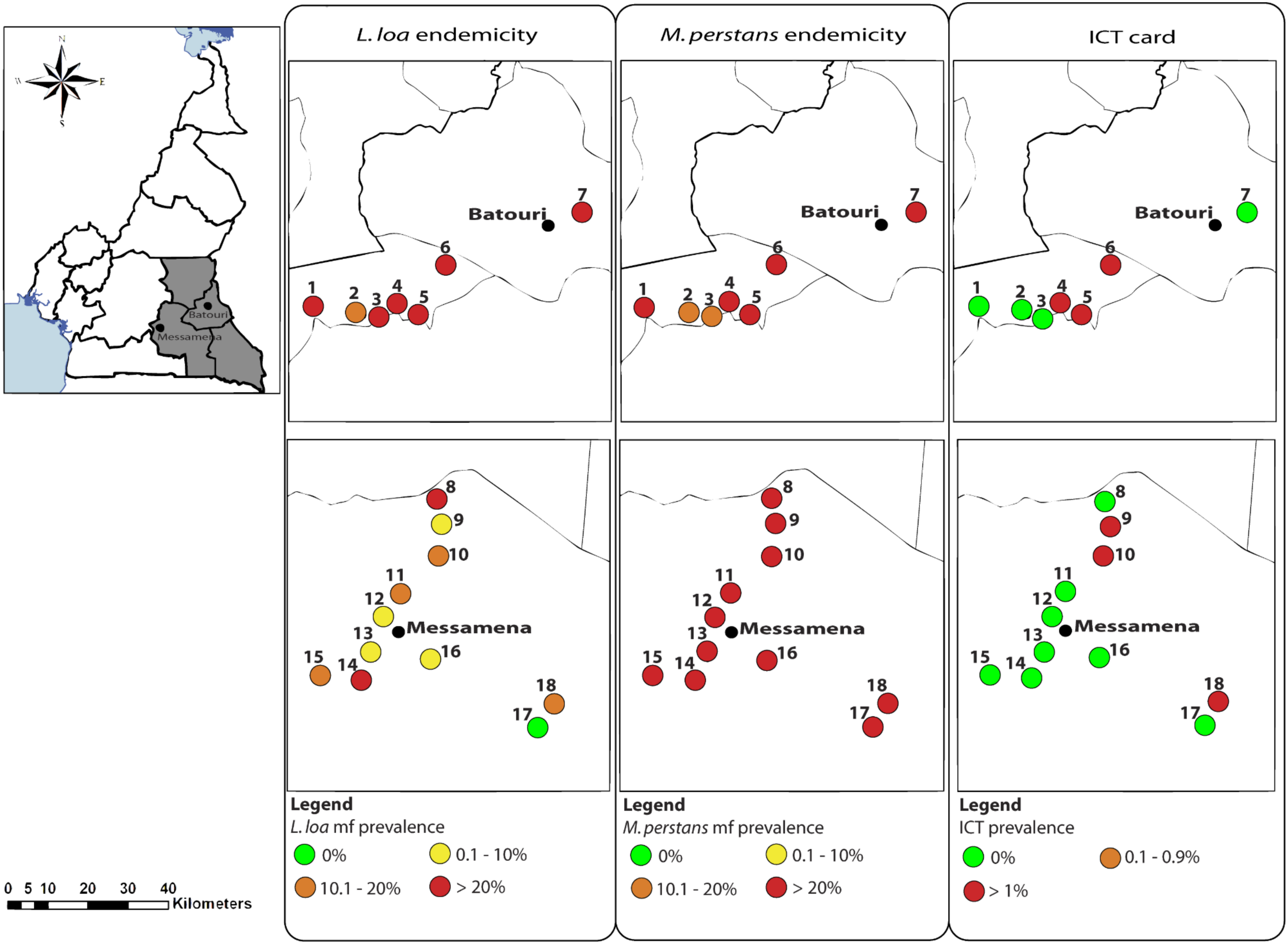
N
S
W
E
Batouri
Messamena
0 5 10 20 30 40
Kilometers
L. loa endemicity
M. perstans endemicity
ICT card
Batouri
Messamena
Legend
L. loa mf prevalence
0%
0.1 - 10%
10.1 - 20%
> 20%
Legend
M. perstans mf prevalence
0%
0.1 - 10%
10.1 - 20%
> 20%
Legend
ICT prevalence
0%
0.1 - 0.9%
> 1%
