# Supplementary figures and images for: Cross-Reactivity of Filariais ICT Cards in Areas of Contrasting Endemicity of Loa loa and Mansonella perstans in Cameroon: Implications for Shrinking of the Lymphatic Filariasis Map in the Central African Region
Source: PLoS Negl Trop Dis. 2015 Nov 6;9(11):e0004184. doi: 10.1371/journal.pntd.0004184 (PMC4636288; doi:10.1371/journal.pntd.0004184)

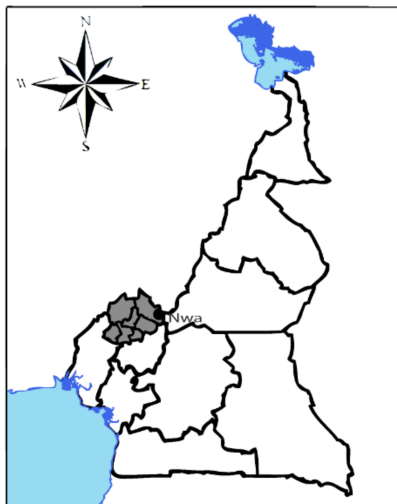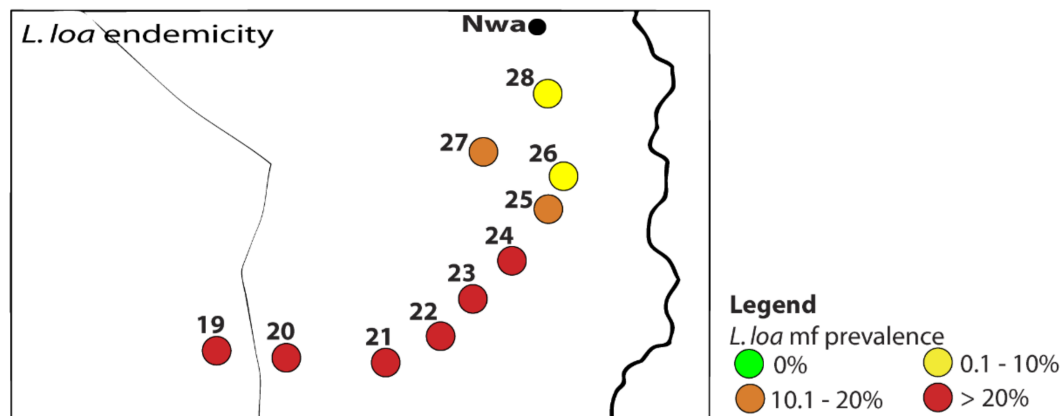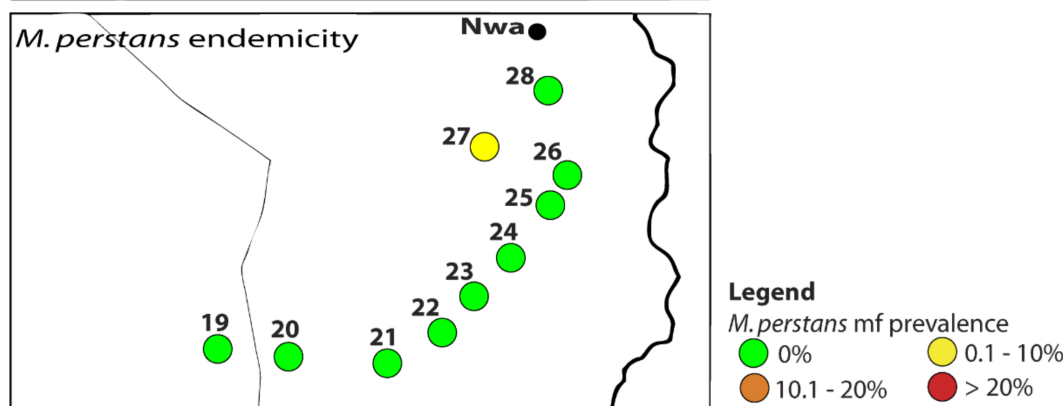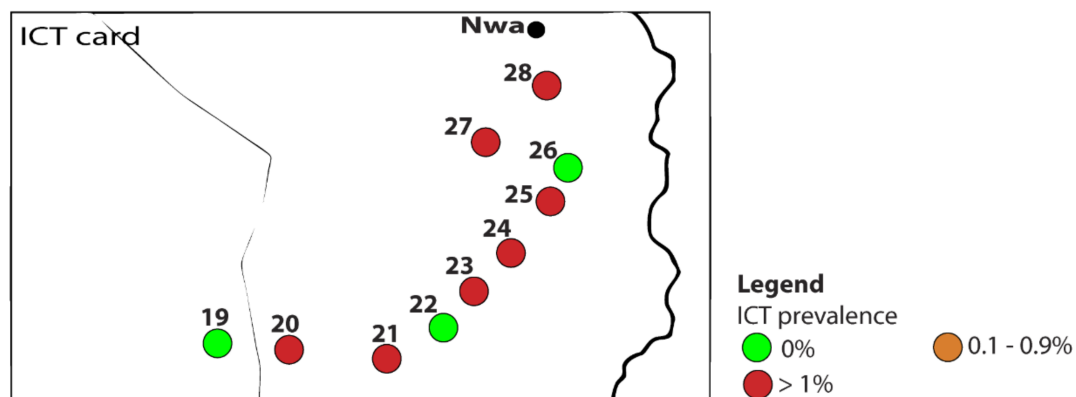

0 1.5 3 6 9 12 Kilometers

Supplement: S2 Fig — 19. Ntem, 20. Nwanti, 21. Nguri, 22. Ngu, 23. Nking, 24. Mbiripkwa, 25. Nwat, 26. Sabongari, 27. Jator, 28. Ngomkow. (PDF) [file pntd.0004184.s003.pdf]

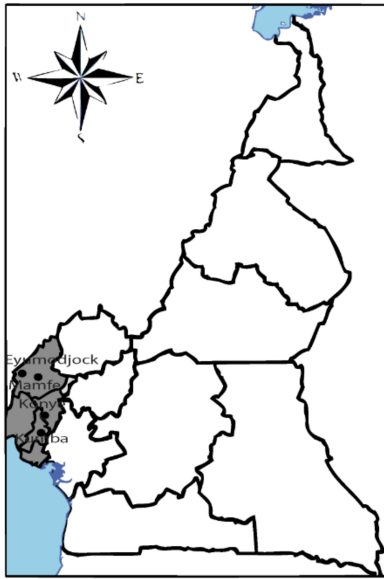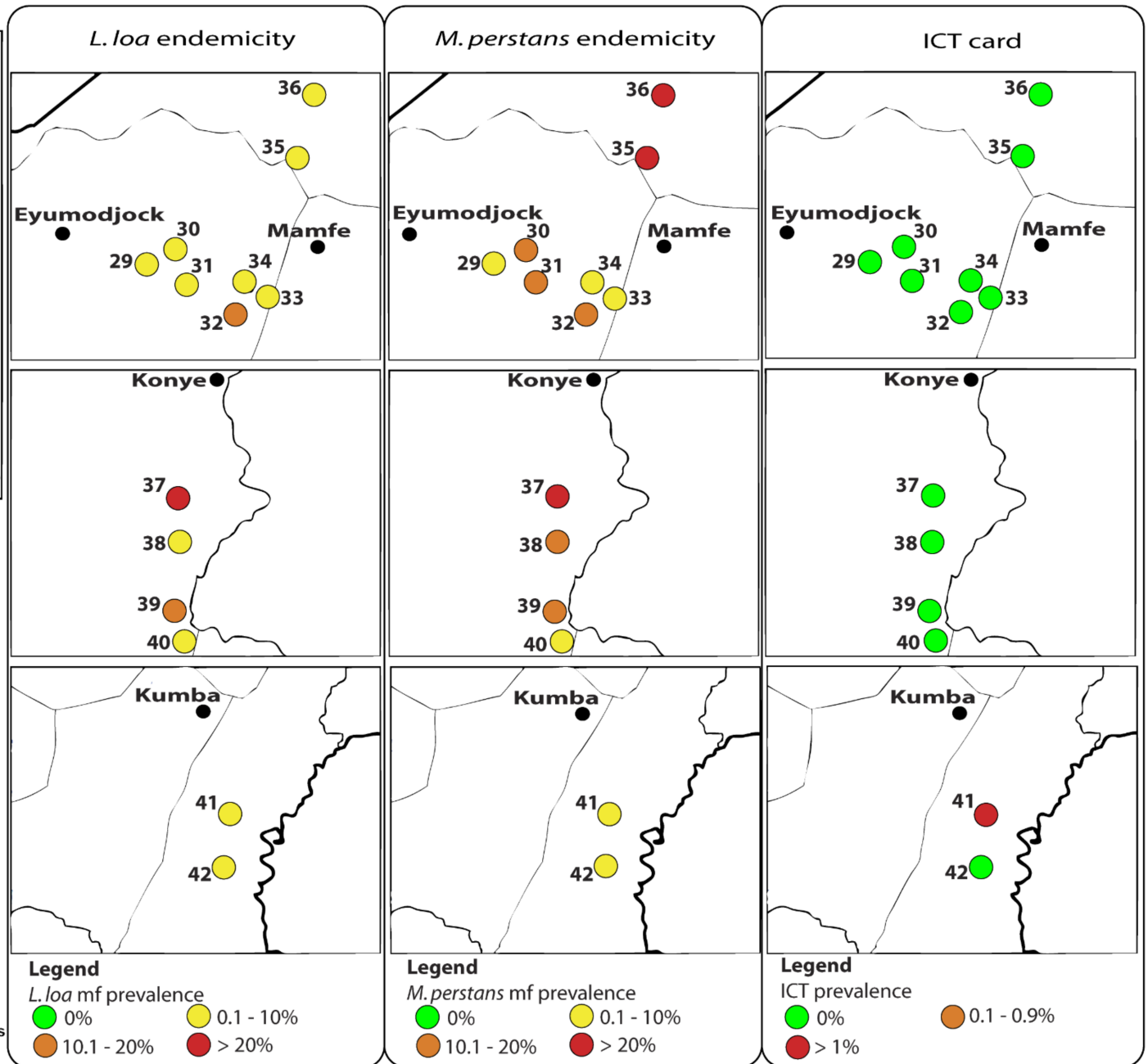

0 4 8 16 24 32 Kilometers

Supplement: S3 Fig — 29. Mbakem, 30. Taboh, 31. Ayukaba, 32. Mbatop, 33. Eyanchang, 34. Ebam, 35. Kesham, 36. Bache, 37. Weme, 38. Bolo, 39. Baduma, 40. Matondo, 41. Ediki, 42. Mbalangi. (PDF) [file pntd.0004184.s004.pdf]
